# RAISE: A Management and Organizational Sustainability Tool for Local Governments to Systematically Self-Evaluate the Effectiveness of Their Programs

**DOI:** 10.1097/PHH.0000000000001515

**Published:** 2022-07-22

**Authors:** Maheen Malik, Kate Graham, Laura O'Donnell, Paul Nyachae, Denis Sama, Mary Carmen Reid

**Affiliations:** Bill & Melinda Gates Institute for Population and Reproductive Health, Johns Hopkins Bloomberg School of Public Health, Baltimore, Maryland (Dr Malik and Mss Graham, O'Donnell, and Reid); and Jhpiego, Nairobi, Kenya (Messrs Nyachae and Sama).

**Keywords:** family planning, local governments, management, self-reliance, tool

## Abstract

Many donor-driven public health programs are now seeking evidence for sustainability prior to investment, creating the need for tools to better appraise these capabilities. Assessing the sustainability of programs and interventions at the local level remains a community-wide challenge.

**Program:**

This article presents a new self-assessment tool, the Reflection and Action to Improve Self-reliance and Effectiveness (RAISE) Tool (“the Tool”), modeled after The Challenge Initiative's (TCI) Sustainability Pillars. It describes the evolution of the Tool, explores its structure and applications, demonstrates its data analysis capabilities, and illustrates how it can be used for continuous program self-assessment by local governments, which TCI considers an indicator of program sustainability at the local level.

**Implementation:**

Developed in 2019, the Tool has been adapted, adopted, and implemented by 92 local governments across 11 countries. The Challenge Initiative works with these local governments over a minimum of 3 years, providing management and technical coaching on high-impact interventions. Using the Tool, local governments self-assess and evaluate the quality and effectiveness of their activity implementation and identify gaps for improvement. The Tool helps both local governments and TCI track their readiness toward becoming self-reliant and taking ownership of their family planning programs.

**Evaluation:**

As of June 30, 2021, 39 of the 92 local governments reached the final stage of maturity, self-reliance.

**Discussion:**

Experts have stated that it can take 15 years for a sustainability assessment tool such as RAISE to be adopted into government policies. After 2 years of using the Tool on a quarterly basis, on average 87.3% of eligible local governments completed the self-assessments, made course corrections, and have taken steps toward program independence. The 39 local governments that successfully progressed to self-reliance continue to use the Tool without TCI's coaching support and have expressed interest in adapting the Tool for other health interventions.

## Context

Sustaining impactful health programs is critical to maintaining expected program benefits, strengthening community-wide capacity, and optimizing available resources.^1^ For decades, the development community and donors have strived to improve government systems for increased health impact in sustainable ways. Too often, projects and investments are structured and implemented in ways that are reliant on ongoing donor support to sustain health outcomes, resulting in persistent concern for the longevity of skill and training heavy health interventions following program defunding.^2^ When external support ends at the closeout of a project, changes made and improved health outcomes often fall back to preproject levels. Not all programs are sustained beyond their initial implementation period partly due to uncertainty regarding sustainability, including infrequent or inappropriate use of sustainability assessments.^3^ Determining whether programs can be sustained, and whether there is commitment on behalf of local governments and health systems to consistently monitor such programs, is critical in ensuring long-term, systemic changes.^4^

There are societal and systems challenges that cannot be easily fixed, including complex administrative systems, health care worker strikes, political elections, and supply chain management issues.^5^ However, there was a need to develop a standardized method of determining government capacity and the institutionalization of programs that provides consistent results that are comparable across time and location. The Challenge Initiative (TCI) is a program led by the Bill & Melinda Gates Institute for Population and Reproductive Health in the Department of Population, Family and Reproductive Health at the Johns Hopkins Bloomberg School of Public Health. The Challenge Initiative, with its “business unusual” approach to financing, scaling up, and sustaining reproductive health solutions in urban poor areas in Africa and Asia, stepped up to address this challenge. The Challenge Initiative created and implemented the Reflection and Action to Improve Self-Reliance and Effectiveness (RAISE) Tool (“the Tool”)^6^ that measures progress toward sustainability of local government health programs.

The Challenge Initiative applies evidence-based family planning and adolescent and youth sexual and reproductive health (FP/AYSRH) interventions related to service delivery, demand generation, and advocacy, and helps local governments to right-fit them for their context. The Challenge Initiative's curated tools and high-impact approaches are designed to help local governments implement their own evidence-based FP/AYSRH interventions. The Challenge Initiative provides technical and management coaching to local governments, empowering them to rapidly and sustainably scale up interventions in urban areas. The Challenge Initiative does not implement FP/AYSRH programs directly. Instead, local governments at the city, state, or county level self-select to partner with TCI. They bring financial and political commitment to lead—and own—the effort, while TCI provides ongoing coaching and technical guidance in return, as the local governments implement proven FP/AYSRH interventions and work toward self-reliance. Selection criteria typically include some level of system readiness of each participating city. A typical engagement period of direct TCI support is approximately 3 years. Implementation of these high-impact interventions (HIIs) and self-assessments continue even after TCI direct coaching support ends. The Challenge Initiative is currently being implemented in 11 countries across 5 regional hubs: East Africa, Francophone West Africa, India, Nigeria, and the Philippines.^7^ The Challenge Initiative's accelerator hub partners include Johns Hopkins Center for Communication Programs, Population Services International, Jhpiego, IntraHealth International, and Zuellig Family Foundation.

### The Raise Tool

The Tool was developed to assess the quality, implementation strength, and sustainability of a TCI partner city's FP/AYSRH program. The Tool is meant to be used directly by local government staff to foster ownership and commitment toward achieving sustained FP/AYSRH programs. The results help identify performance levels of sustainability components that are critical in a city's journey to self-reliance and provide a basis for coaching on continuous improvement. The Challenge Initiative provides coaching and technical assistance to local governments as they move through the RAISE assessments, integrating approaches from TCI University, an online platform that houses TCI's high-impact urban FP/AYSRH interventions for learning, adapting, disseminating, and coaching.^7^ Activities key to TCI's early achievements in each city, county, and state include measuring the intensity of FP/AYSRH program implementation, implementing course corrective actions, maintaining quality during rapid scale-up, encouraging local ownership that leads to sustainability, and engaging local governments and other stakeholders in decision making in meaningful ways. The Tool utilizes a standard set of indicators that consider all these elements and help governments reflect on their implementation progress in 4 main areas, identified as TCI's Sustainability Pillars.

### Sustainability Pillars, the Framework of RAISE

**FIGURE 1:**
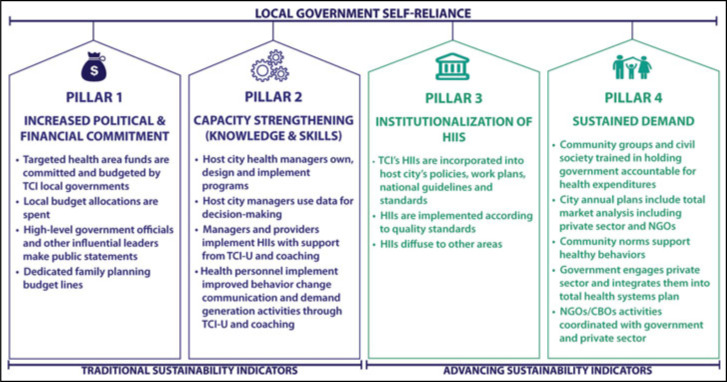
The Challenge Initiative's (TCI's) Sustainability Pillars Abbreviations: CBOs, community-based organizations; HIIs, high-impact interventions; NGOs, nongovernmental organizations; TCI-U, The Challenge Initiative University. This figure is available in color online (www.JPHMP.com).

Sustainability is a key topic of importance in public health, and many have tried to determine how programs successfully build systems that result in sustained improvements in health outcomes.^8^ The Challenge Initiative realized its potential to not only affect the uptake of FP/AYSRH services but also improve local health systems through TCI's coaching and presence in the cities. The Challenge Initiative identified 4 pillars to measure movement toward sustainable scale-up of high-quality FP/AYSRH programming and outcomes (Figure 1). These pillars represent TCI's thinking on predictors of sustainability; the indicators that help signal a city's progress toward self-reliance. Using the pillars as a guide, TCI more meaningfully captures system changes resulting from TCI's efforts to institutionalize proven approaches at all levels of the health system, build sustained demand, and improve attitudes and behaviors toward FP/AYSRH.

Pillars 1 and 2 are common measures of sustainability—increased political and financial commitment and strengthened capacity in health programming and service delivery. Pillar 3 seeks to capture systemic changes resulting from TCI's approaches. The proven approaches must be institutionalized at all levels of the health system to ensure that they are sustained. This is a core indicator of success of the TCI business model. Pillar 4 is sustained demand for health services. This includes improved community norms that support healthy behaviors and the engagement and involvement of all stakeholders in health systems. The private sector, nongovernmental organizations, and faith-based organizations in most countries deliver a significant proportion of the health care. Civil society and community members have an important role to play in monitoring and holding governments accountable for delivering accessible, affordable, and high-quality health care. Governments need to coordinate all of these stakeholders to ensure sustainable and improved health outcomes. These 4 Sustainability Pillars signal a local government's progress toward greater self-reliance and thus became the domains on which the RAISE Tool is based (see Supplemental Digital Content Table 1, available at http://links.lww.com/JPHMP/A937).

For the purposes of this article, TCI uses sustainability and self-reliance interchangeably.

### Development of the Tool

The Challenge Initiative reviewed several external documents and sustainability tools, including the Management and Organizational Sustainability Tool, NuPITA project's Organizational Capacity Assessment Tool, and MSI's Capacity Scan (CAPScan) diagnostic tool. These tools were aligned to TCI's internal results framework to develop the first internal draft of the Tool in 2019.^9^–^11^ After several rounds of consensus meetings with program implementation experts, coaches, FP/AYSRH technical staff, city managers drawn from TCI, and select local government health management teams with high-impact FP/AYSRH experience, the Tool was pretested across 10 locations in Nairobi, Kenya. The Challenge Initiative incorporated feedback and a second pretest was rolled out in 7 locations across Uganda and Tanzania. Again, TCI incorporated their feedback and rolled out the Tool more widely to 47 TCI-supported local governments across Kenya, Tanzania, and Uganda. In early 2020, TCI addressed feedback from pretesting and implementation, subsequently introducing the Tool to TCI-supported local governments in Nigeria, India, and Francophone West Africa. Each regional hub field tested the Tool in a few locations with key government stakeholders, whose input was integral to inform country-specific versions of the Tool that fit their local context. By June 2020, hubs began using the adapted version of the Tool in all eligible locations. The Tool was developed after many TCI partner cities had already been implementing FP/AYSRH programs for 1 to 2 years, so it should be noted that cities had received varying levels of coaching and technical assistance from TCI by the time they began using the Tool. East Africa began implementing the Tool first in 2019 therefore it has completed 7 RAISE assessment rounds followed by Francophone West Africa (2019), Nigeria and India (June/July 2020) who have completed 4 to 5 rounds, respectively. Because Philippines began using the Tool most recently (2021) and has only completed 1 round of the assessment, they are excluded from the data in this article.

### Tool Usage and Structure

The Tool was designed to be used by local government teams responsible for implementing FP/AYSRH programs, though the Tool can be adapted for health areas outside of FP/AYSRH. All hub partners, in coordination with their respective local governments, adapted the Tool to meet their needs. The Challenge Initiative encouraged a diverse group of participants to participate in the RAISE assessment process, including technical leads (eg, district medical officers, FP coordinators), health information staff, finance staff, political leaders (eg, mayors, local health ministers), advocacy core group members, FP technical group members, private sector key stakeholders, and TCI focal staff. This approach was intended to bring different perspectives on common issues, foster objectivity, and facilitate action planning (see Supplemental Digital Content Table 2, available at http://links.lww.com/JPHMP/A938).

**FIGURE 2:**
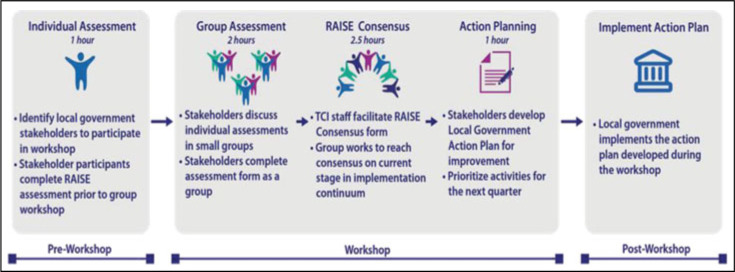
The Challenge Initiative Description for the Process of Conducting Assessments Using the RAISE Tool Abbreviations: RAISE, Reflection and Action to Improve Self-Reliance and Effectiveness; TCI, The Challenge Initiative. This figure is available in color online (www.JPHMP.com).

Utilization of the RAISE Tool takes place over 1 full working day, or 2 half days. The **preworkshop** is the first step, wherein participants complete RAISE assessments individually. This allows participants to document their personal reflection and thought, rather than relying on groupthink for scoring (Figure 2). A participatory **workshop** is then held, where participants come together to provide and discuss their feedback in small working groups. Working groups reach consensus on the scoring of the different indicators and on the final score, providing evidence for their scoring rationale. A final score for the city is calculated on the basis of scores for each assessment area. Scores and levels of performance are defined in Table 1. Groups also highlight course corrections that should be made in areas that are affecting their readiness toward self-reliance. Together, participants develop an action plan with clear roles and responsibilities assigned to each stakeholder, and deliverables and timeline outlined. **Postworkshop,** the action plan is implemented and monitored. This assessment process is conducted quarterly during a city's active engagement with TCI, transitioning to biannually once that city reaches the self-reliance stage.

**FIGURE 3:**
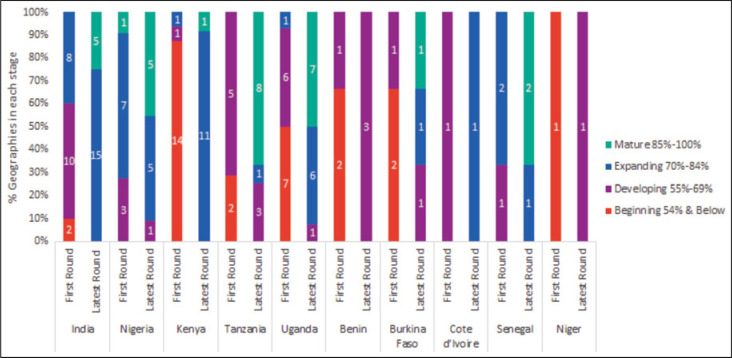
First and Latest Round of RAISE Assessments Across Countries With at Least 2 Rounds of RAISE

**TABLE 1:** Four Levels of Performance During RAISE Assessment

Stage	Score	Description of Score
Beginning	54% and less	Local government's development domains, systems, and processes are at minimum and there is a need for focused and intensive coaching support to improve and increase the capacity of the domains.
Developing	55% to 69%	Local government has basic level of capacity. While all basic systems and processes are in place, select domains have ongoing weaknesses and there is a need for continued coaching and strengthening of the identified domains.
Expanding	70% to 84%	Local government can rapidly respond to change and sustain itself due to credible systems, adequate resources, and viable family planning programs. As the government is managing its program more smoothly, TCI reduces the coaching support it provides to these governments as they are on their way to sustaining the program.
Mature	85% to 100%	Local government has well-developed and well-functioning credible systems, adequate resources, and viable family planning programs. The city has moved along the continuum to becoming self-reliant. Teams have reported improvements in accountability and overall management of FP/AYSRH programs. Cities have reached self-reliance.

Abbreviations: FP/AYSRH, family planning and adolescent and youth sexual and reproductive health; TCI, The Challenge Initiative.

For the purposes of this article, the self-assessment scores by each country adopting the tool will not be discussed. This article instead focuses on how successful quarterly self-assessment leads to self-reliance.

Of the 111 local governments implementing TCI's HIIs, 92 have consistently undertaken RAISE assessments and used their results and postworkshop action plans to course correct and to address their identified gaps by indicator and domain (see Supplemental Digital Content Table 1, available at http://links.lww.com/JPHMP/A937). As of the latest round in June 2021, 39 locations (16 in India, 3 in Nigeria, and 20 in East Africa, representing 43.3% of those consistently completing RAISE assessments) have transitioned to self-reliance (“Mature” stage). All 39 locations have institutionalized the Tool into their planning and have continued completing assessments every 6 months. Self-reliance scores monitored after these locations transitioned to the “Mature” stage indicate that they have been able to maintain the gains made during their active engagement with TCI. Forty-seven additional locations improved their performance in the 4 domains: political and financial commitment, capacity strengthening, institutionalization, and sustained demand and, using the same methodology, have transitioned from the “Developing” to “Expanding” stage.

Figure 3 demonstrates countries' progress made toward self-reliance over 3 to 4 assessment rounds. This figure is included not to highlight specific data points, but rather as evidence that countries are regularly using the Tool for self-assessment and future project planning. It should also be noted that there are small differences in totals in East Africa as more geographies came on board and some became self-reliant by round 7.

### Data Dissemination and Uses

Information generated from the RAISE data is disseminated on a quarterly basis to TCI program staff, local governments, political leadership, and other stakeholders through government meetings, coaching and mentorship events, and formal reports. These data are used by both TCI and local governments to understand the level of implementation quality and sustainability of TCI-supported interventions and develop their action plans. They are also used for best practice sharing, focused coaching and training of local government implementers, and fostering a sense of ownership by local governments. Results help local governments to modify their own coaching plan and ensure that coaching trickled down to other stakeholders includes not only coaching on HIIs but also management coaching.

### Defining and Enabling Self-Reliance

The Challenge Initiative envisions self-reliance as an environment wherein local governments can effectively manage and sustain FP/AYSRH programs. They have successfully improved programming and coordination between departments, implementing partners, and donors, and not as an exit strategy or one-time event. This shift to self-reliance is a time for the local governments to celebrate. In TCI's experience, it takes local governments 3 to 3.5 years to reach self-reliance.

There is often a misnomer that self-reliance means donor independence, and that local governments no longer require funding or support. They may continue to require some support. The point to emphasize is that they are now the leaders and drivers of their programs and are able to connect the dots and run their own FP/AYSRH programs, coordinate with implementing partners and donors to avoid duplication, gradually increase local contributions toward FP/AYSRH interventions, and ensure that the funding that has been earmarked for these interventions is released.

Specific milestones have been set forth to determine where a local government is in terms of self-reliance based on their RAISE assessment scoring. Cities must show increasing RAISE scores to consider themselves prepared for self-reliance (a score of 85% and above). Institutionalizing the Tool into government systems also weighs into discussions about self-reliance, as users have expressed that the Tool is seen as an important component of the sustainability of strengthened health systems. It has proven to maintain the level of impact that was achieved during a local government's active engagement with TCI.

## Methods

For the purposes of this study, we examined the adoption of the Tool by 92 local governments to understand the effectiveness of a self-assessment tool in support of public health initiatives.

The authors did not request institutional review board approval as this research qualified as exempt, per **§46.104 Exempt research, Section 4, iv**. Consent is not required, nor is any identifiable private information collected or used. The research presents no more than minimal risk of harm to subjects and involves no procedures for which written consent is normally required outside of the research context.

### Data collection

Of the 111 local governments implementing, 92 were eligible to undertake RAISE assessments using the Tool (81.1% adoption rate). This article reports on the quarterly self-assessment completion rate by the 92 eligible local governments, and how completion affects self-reliance. Each country should complete 4 rounds per year to meet the quarterly design. The results of use, and the impact toward self-reliance, are reported on the basis of completion rates by quarter and total quarters eligible for completion.

## Results

This analysis looked at the 2 years of Tool use from across 92 local governments to help determine whether an association exists between consistent use of the Tool and health system changes. A positive association is expected and would indicate that the Tool is aiding TCI partner cities in reaching self-reliance and institutionalized changes within their health systems.

The East Africa hub, having begun using to implemented the Tool 1 year prior to other hubs, has completed 7 of a possible 7 rounds (100%) of RAISE assessments with 47 local governments across Tanzania, Uganda, and Kenya. On average, across 7 rounds of RAISE, 86.2% of local governments eligible to undertake RAISE assessments have completed them on a quarterly basis. Francophone West Africa began rolling out the Tool in the last quarter of 2019 and completed 5 rounds out of 6 possible rounds (83.3%) with 12 local governments across Benin, Burkina Faso, Cote d'Ivoire, Niger, and Senegal. More than 78% of those local governments, on average, completed quarterly assessments. Nigeria and India began using RAISE in June and July 2020 and have both completed 4 rounds. In Nigeria, of the 13 eligible states, 95.6% consistently completed quarterly assessments. In India, 98.7% of local governments (n = 20) in Uttar Pradesh state completed quarterly assessments.

It should be noted that due to COVID-19, some cities were unable to gather for consensus meetings and complete the Tool every quarter due to nonavailability of staff who were assigned to COVID-19–related duties. However, for most cities, RAISE and group consensus meetings continued virtually.

The data generated from the Tool have also been used to measure the extent of achievement of various quality- and sustainability-related indicators and local government's progress toward self-reliance. Overall, there was a consistent increase throughout most locations (Table 2). The Tool has been helpful in showing how the COVID-19 pandemic affected other areas of the health system, as certain indicators within the domains were impacted and reflected in RAISE assessments.

**TABLE 2:** Average RAISE Scores for Cities That Implemented Public Health Programs, by Round and Region From 2019 to 2021

Location and Total Number of Geographies	RAISE Round	Number of Geographies Completing RAISE During the Period	Total Number of Geographies Eligible to Complete RAISE Assessment	Completion Percentage of Eligible Geographies	Average RAISE Scores per Hub
East Africa—47 total geographies	1	38	38	100%	56%
	2	39	39	100%	70%
	3	30	42	71.4%	76%
	4	27	43	62.8%	76%
	5	34	43	79.1%	80%
	6	45	47	95.6%	80%
	7	39	40	97.5%	83%
Overall average				**86.2%**	**74%**
Francophone West Africa—12 geographies	1	11	12	91.7%	55%
	2	7	12	58.3%	61%
	3	9	12	75%	64%
	4	9	12	75%	69%
	5	11	12	91.7%	75%
Overall averages				**78.3%**	**65%**
India—20 geographies	1	20	20	100%	68%
	2	20	20	100%	73%
	3	19	20	95%	76%
	4	20	20	100%	80%
Overall averages				**98.7%**	**74%**
Nigeria—13 geographies	1	11	11	100%	77%
	2	11	11	100%	80%
	3	11	11	100%	83%
	4	11	13	84.6%	83%
Overall averages				**95.6%**	**81%**

Abbreviation: RAISE, Reflection and Action to Improve Self-Reliance and Effectiveness.

Of the 39 local governments that have progressed to self-reliance, East Africa and India represent the majority (92.3%; 16 in India, 3 in Nigeria, and 20 in East Africa) of geographies. Those that have become self-reliant have also more consistently completed the Tool on a quarterly basis. Although it is still early in Tool adoption and implementation, the data points toward those that consistently complete rounds of the assessment are more likely to become self-reliant, meaning that those local governments are consistently monitoring their FP/AYSRH programs, identifying gaps and solutions, and making course corrections.

## Discussion and Conclusion

Sustainability and assessing the sustainability of local government programs are critical to ensuring health system changes. It has been noted that there is a need for tools to systematically assess governments' readiness toward self-reliance.^6^ By developing a Tool to assess “maturity” of self-reliance, TCI facilitated a systematic way to evaluate and track progress over time. The self-assessment demonstrates to the local government the maturity of their program, enabling local ownership over goal setting. In this way, the Tool functions as evidence-based, unique to implementing regions as they iteratively identify and meet specific needs when moving toward self-reliance. Using TCI's Sustainability Pillars framework to identify gaps for improvement, local governments can make timely course corrections and move toward programmatic self-reliance and readiness for program independence—also indicating preparedness for eventual self-monitoring. By targeting gaps in self-sufficiency, the Tool helps tailor coaching and technical assistance to address identified gaps. The Tool is unique in that it is flexible, adaptable, and resilient to change. Experience has so far demonstrated that local governments are willing to adapt and adopt this Tool, as, on average, 88.2% of eligible local governments across East Africa, Nigeria, Francophone West Africa, and India completed quarterly assessments consistently.

There are several challenges that need to be addressed in the years ahead in order for the Tool to catalyze sustainable improvements. The Tool provides qualitative data to measure local governments' progress. However, TCI has yet to fully triangulate the RAISE data with quantitative data, such as contraceptive uptake, from governments' health management information systems (HMIS). Triangulation will help TCI and its government partners begin to analyze the association between changes in RAISE scores, which indicate system strengthening, and changes in contraceptive uptake. Doing so would help determine whether the Tool is aiding TCI partner cities in increasing their contraceptive uptake. In addition, ensuring that local governments prioritize use of the Tool over other available tools is a challenge. As Larry Cooley, Founder and President Emeritus of Management Systems International, said in the foreword to the RAISE Tool document, the process of fully institutionalizing a new tool such as RAISE into the government system can take 12 to 15 years before it becomes the “new normal.”^6^

The Tool has been helpful for local governments to compare each program's current sustainability status to when they began implementing. With positive impacts already being seen, local governments are noting that the “the Tool helps us to be reminded about what we always forget during work planning and it will help us during work plan review ... but now that they are captured in the Tool, we have assigned a specific person to follow them up (HMIS Focal Person, Kira Municipality, Uganda).” This process also visually represents the progress made on a quarterly basis, which further incentivizes governments to want to continue to better their health systems and reach the goals that they have outlined. The Challenge Initiative uses self-reliance as a point of pride for governments and sets them up as an example to others for how to make the best use of the resources given, while ultimately managing to instill new best practices that will continue beyond the project.

The success of this Tool is not that the Tool does the work. It is that the Tool serves as a reminder and template for self-assessment empowering governments to set goals, identify gaps, self-assess, and form new goals and action plans. This cycle of program improvement demonstrates the sustainability afforded to government programs, which is an important first step to meaningful systemic adaptation. While increasing scores demonstrate how countries' self-assessment has moved them toward self-reliance, future research is necessary to better understand these data.

**Implications for Policy & Practice**

- The Tool's assessments and results have proven useful in providing critical reminders to local government staff of the action items they need to complete, including a clear plan identifying who is responsible for each item and the resources required. These action plans facilitate advocacy and have enabled local governments to obtain additional resources to support program implementation. The RAISE process disrupts silos and vertical programs by ensuring departments work collaboratively and supportively. Political leaders and advocates have been able to support health teams in championing and financing program scale-up.
- Including the RAISE process in the work plans of local governments will help enhance the practice of tracking progress of self-reliance and sustainability of HIIs. Local governments have used this to provide incentives for performing well, offering recognition among their peers. This gives stakeholders greater motivation and fosters healthy competition between cities. As local governments use this to measure system strengthening, the Tool has the potential to be adapted for use by other health areas.
- Incorporating data from other sources, such as HMIS, project management information systems, and qualitative studies into the assessment process, RAISE provides a complementary and holistic view of progress toward self-reliance and program accomplishments.

## Supplementary Material
