# Supplementary material for: RAISE: A Management and Organizational Sustainability Tool for Local Governments to Systematically Self-Evaluate the Effectiveness of Their Programs
Source: J Public Health Manag Pract. 2022 Jul 22;28(5):550–8. doi: 10.1097/PHH.0000000000001515 (PMC9439692; doi:10.1097/PHH.0000000000001515)
Supplement: Supplementary file 1 [file jpump-28-550-s001.docx]

| Domain | Component | Criteria | Rating (1-4) | | Selected FP Score | FP Evidence | Selected AYSRH Score | AYSRH Evidence |
| --- | --- | --- | --- | --- | --- | --- | --- | --- |
|  |  |  | FP | AYSRH |  |  |  |  |
| Domain 1 | Political and Financial Commitment for FP Intervention | | | | | | | |
| 1.1 | **Local Government Mission Statement**  *Review the mission statement, its availability, knowledge by staff, reference and how FP/AYSRH programs contribute towards it.* | Mission statement is not available or, if there is one, it is not posted where staff and/or visitors see it regularly (i.e., notice boards, strategic places and walls, etc.). | **1** | **N/A** | **1** |  | **N/A** |  |
|  |  | Mission statement is posted where staff and/or visitors see it regularly. | **2** | **N/A** |  |  |  |  |
|  |  | Mission statement is known by staff and stakeholders at all levels of health service delivery. | **3** | **N/A** |  |  |  |  |
|  |  | Mission statement is, known and frequently referred to in government materials (i.e., handbooks, strategic and operational plans, etc.) and FP programs contribute to it. | **4** | **N/A** |  |  |  |  |
| 1.2 | **Local Government Values**  *Review the values, availability, knowledge, reference and adherence by staff.* | Values are not posted where staff and/or visitors see them regularly (i.e., notice boards, strategic places and walls, etc.) | **1** | **N/A** | **1** |  | **N/A** |  |
|  |  | Values are posted where staff and/or visitors see them regularly | **2** | **N/A** |  |  |  |  |
|  |  | Values are known and frequently cited by staff and stakeholders at all levels of health service delivery | **3** | **N/A** |  |  |  |  |
|  |  | Staff are held accountable for adhering to the values | **4** | **N/A** |  |  |  |  |
| 1.3 | **Advocacy for FP/ AYSRH**  *Assess the extent of advocacy and engagement of policy makers to advance the FP needs of the population.* | High-level government officials and other influential leaders are passively, or not advocating for FP/AYSRH in the community. | **1** | **1** | **1** |  | **1** |  |
|  |  | High-level government officials and other influential leaders are actively advocating for FP/AYSRH in the community | **2** | **2** |  |  |  |  |
|  |  | High-level government officials and other influential leaders are making public statements at forums, in media, public events, etc., and visibility is high. | **3** | **3** |  |  |  |  |
|  |  | FP/AYSRH issues and priority needs of the community are included in the agenda of high-level LG meetings. LG has adopted and/ or supported policies (laws, regulations, budgets, etc.) that advance FP/AYSRH needs of the community | **4** | **4** |  |  |  |  |

Figure 7: Excerpt of RAISE scoring guidelines
