# Supplementary material for: RAISE: A Management and Organizational Sustainability Tool for Local Governments to Systematically Self-Evaluate the Effectiveness of Their Programs
Source: J Public Health Manag Pract. 2022 Jul 22;28(5):550–8. doi: 10.1097/PHH.0000000000001515 (PMC9439692; doi:10.1097/PHH.0000000000001515)
Supplement: Supplementary file 2 [file jpump-28-550-s002.docx]

| **East Africa** | **Francophone West Africa** | **India** | **Nigeria** |
| --- | --- | --- | --- |
| - Reproductive health coordinator - AYSRH coordinator - Community mobilization coordinator - HMIS coordinator - Youth representatives - Private sector representatives - Town clerks - Health center in charge - Director Health - Political leadership representative - Logistics and medicines coordinator - Health educator - Finance officer - Planning officer | - Mayor - The President (local elected) of the Health Commission of the City Council - Municipal Secretary - Administrative and Financial Managers - Commission Health system - District Chief Medical Officer - The Reproductive Health Coordinator of the Medical Region - The District Reproductive Health Coordinator - Representatives District Community Health Promotion Actor - SSRAJ focal point of the Medical Region - The manager of the health district - Head of Education Information Health Promotion - The representative of the Communal Youth Council - The representative of the Teen Council Center - Representatives of the Basic Community Organizations (CBOs) - Representatives of religious associations (Imams and parish priest) - Academy Inspection/Education and Training Inspection - Medical Inspection of Schools (IME) - Technical and Financial Partners (TFP) | - Chief Medical Officer (CMO) - ACMO/Nodal Family Planning, Nodal Urban Health - ARO - District Program Managers (DPM) - DAM - DCPM - CCPM (certified clinical project manager) - Divisional /District Urban Health Coordinator - DCAA - FP Logistics Management Information System (FPLMIS) Officer - Medical Officer in Charge - HMIS Coordinator | - Honorable Commissioner for Health - Executive Secretary, State Primary Health Care Development Agency - Director, Public Health - Director, Planning, Research & Statistics - Director, Community Services - State Reproductive Health Coordinator - State Family Planning Coordinator - State Health Educator - State M&E Officer - State Logistics Officer - Adolescent Health Desk Officer - State Finance focal person |

Figure 6: Key stakeholders in RAISE assessment process.
